# Association between omentin-1 expression in human epicardial adipose tissue and coronary atherosclerosis

**DOI:** 10.1186/s12933-016-0406-5

**Published:** 2016-06-28

**Authors:** Yu Du, Qingwei Ji, Lun Cai, Fangjiong Huang, Yongqiang Lai, Yue Liu, Jianbo Yu, Bo Han, Enjun Zhu, Jinwei Zhang, Yujie Zhou, Zhijian Wang, Yingxin Zhao

**Affiliations:** Beijing Institute of Heart Lung and Blood Vessel Diseases, Beijing, 100029 China; Department of Cardiology, Beijing Anzhen Hospital, Capital Medical University, Beijing, 100029 China; Department of Cardiac Surgery Center, Beijing Anzhen Hospital, Capital Medical University, Beijing, 100029 China

**Keywords:** Epicardial adipose tissue, Omentin-1, Atherosclerosis, Coronary artery disease

## Abstract

**Background:**

Omentin-1, a novel adipocytokine mainly expressed in visceral adipose tissue, has been found to inhibit the inflammatory response and improve insulin resistance as well as other obesity-related disorders. This study investigated the association between omentin-1 expression in human epicardial adipose tissue (EAT) and coronary atherosclerosis.

**Methods:**

Serum samples, and paired biopsies from EAT and subcutaneous adipose tissue (SAT), were obtained from patients with and without coronary artery disease (CAD, n = 28 and NCAD, n = 12, respectively) during elective cardiac surgery. Coronary angiography was performed to identify CAD presence. Serum omentin-1 and adiponectin levels were measured by ELISA. mRNA expression of omentin-1 and adiponectin was detected in adipose tissue by quantitative real-time PCR, and omentin-1 protein expression was evaluated by immunohistochemistry. Correlation and multivariate linear regression analyses were performed to determine the association between omentin-1 expression and clinical risk factors.

**Results:**

mRNA and protein expression of omentin-1 were higher in EAT than paired SAT in patients with CAD and NCAD. Compared with NCAD patients, CAD patients had lower omentin-1 and adiponectin mRNA levels in EAT and serum levels as well as lower omentin-1 protein levels. Among patients with CAD, omentin-1 expression was lower in EAT surrounding coronary segments with stenosis than those without stenosis, in terms of mRNA and protein, whereas adiponectin mRNA level in EAT did not seem to differ between stenotic and non-stenotic coronary segments in CAD patients. In multivariate linear regression analysis, CAD was an independent predictor of EAT omentin-1 mRNA expression (beta = −0.57, 95 % CI −0.89 to −0.24; *P* = 0.001) and serum omentin-1 levels (beta = −0.35, 95 % CI −0.67 to −0.03; *P* = 0.036).

**Conclusions:**

Circulating and EAT-derived omentin-1 levels were reduced in patients with CAD. Omentin-1 expression in patients with CAD was lower in EAT adjacent to coronary stenotic segments than non-stenotic segments.

## Background

Obesity is an important risk factor for cardiometabolic diseases and its prevalence has risen substantially over the last decade [1]. Accumulation of visceral adipose tissue, accompanied by chronic low-grade inflammation, is causally linked to the initiation and progression of multiple obesity-related disorders, including type 2 diabetes mellitus (T2DM), dyslipidemia and atherosclerosis [2, 3]. Epicardial adipose tissue (EAT) has emerged as a novel target for stratification of cardiometabolic risk factors due to its distinctive location, and multifaceted metabolic properties with systemic and local effects. Because EAT is not separated from adjacent myocardium or the coronary artery vascular wall by fascia, paracrine or vasocrine interactions are able to occur [4]. In addition, EAT is an active endocrine organ that expresses and secretes numerous adipocytokines [5, 6]. It has been established that the equilibrium between pro-inflammatory and anti-inflammatory adipocytokine production by EAT is altered in certain pathological conditions [7, 8]. In patients with coronary artery disease (CAD), adiponectin mRNA expression was markedly decreased in EAT, whereas multiple pro-inflammatory adipocytokines mRNA expression, including chemerin, IL-1β, IL-6 and TNF-α, were significantly increased [5, 9]. Thus, it is supposed that, adipocytokines secreted by EAT via both paracrine and vasocrine mechanisms may regulate the process of atherosclerosis [4].

Omentin-1, also known as intelectin-1 [10], is abundantly expressed in human visceral adipose tissue and it has been demonstrated to modulate obesity-related cardiometabolic disorders via anti-inflammatory activity [11]. Lower circulating omentin-1 levels associated with various metabolic risk factors, including elevated blood pressure, increased waist circumstance, dyslipidemia and glucose intolerance [12]. In addition, serum omentin-1 levels were negatively associated with carotid plaque and positively correlated with cardiac autonomic neuropathy in patients with T2DM [13, 14]. Patients with CAD have also been observed to have lower circulating omentin-1 levels compared with those without CAD [15, 16]. Furthermore, it has been reported that serum omentin levels is a significant predictor of cardiovascular events in patients with suspected CAD and heart failure, as well as in hemodialysis patients with subclinical atherosclerosis [17–19]. Although omentin-1 has been identified as a promising biomarker for obesity-related cardiometabolic diseases, the association between EAT omentin-1 expression and coronary atherosclerosis has not been clarified. To address this, human omentin-1 mRNA and protein expression in EAT was evaluated in patients undergoing elective cardiac surgery with and without established CAD.

## Methods

### Subjects

Between July and November 2015, the study enrolled 40 patients who underwent elective cardiac surgery. Invasive coronary angiography was performed to identify CAD, and patients were divided into the CAD (n = 28) or non-CAD (NCAD; n = 12) group. CAD group referred to patients with three-vessel disease, left main disease or two-vessel disease with proximal left anterior descending lesion indicated for off-pump coronary artery bypass grafting (CABG). NCAD group referred to patients undergoing open-heart surgery for valvular replacement or atrial septal defect repair and no stenosis was found in coronary artery lumen. Key exclusion criteria were: age >80 years, acute myocardial infarction, active chronic inflammation disease, liver or renal failure, and pharmacological glucocorticoid or immunosuppressive therapy.

The study protocol complied with the Declaration of Helsinki and was approved by the ethics committee of Beijing Anzhen Hospital of Capital Medical University. Written informed consent was obtained from each patient before enrollment.

### Clinical data collection

Clinical characteristics, including demographic data, body weight, height, waist circumference, medical history and medication use, were obtained from the hospital records. Body mass index (BMI) was calculated as weight (kg) divided by square of height (m^2^).

### Blood sample measurement

After an overnight fast of 12 h, venous blood samples were collected in sodium heparin Vacutainers (Becton–Dickinson), followed by centrifuging for 15 min at 3000×*g*, and storage of serum samples at −80 °C. Baseline levels of fasting glucose, insulin, glycosylated serum protein, lipid profiles, creatinine, and high-sensitivity C-reactive protein (hsCRP) were measured in the central laboratory of Beijing Anzhen Hospital. Homeostasis model assessment of insulin resistance (HOMA-IR) was used to estimate insulin sensitivity, as calculated by fasting glucose (mmol/L) × fasting insulin (μU/mL)/22.5.

Concentrations of serum omentin-1 (BioVendor, Czech Republic) and adiponectin (R&D System, USA) were detected by commercially available enzyme-linked immunosorbent assay (ELISA) kits following the manufacturer’s instructions. ELISA intra-assay and inter-assay coefficients of variation were both <5 %. All samples were measured in duplicate.

### Adipose tissue acquisition

Two portions of adipose biopsy were harvested, including EAT near right coronary artery ostium, with or without local coronary stenosis, and subcutaneous adipose tissue (SAT) from the area of chest incision. The tissue samples (average 0.4 g) were rinsed with phosphate-buffered saline and then divided into two portions. One portion was stored in liquid nitrogen for RNA isolation and the other was immersed in neutralized formalin for immunohistochemistry.

### RNA isolation and quantitative real-time PCR

Total RNA was extracted from samples using Trizol reagent (Invitrogen, USA). The concentration and purity of extracted RNA were assessed by calculating the ratio of optical density at 260 and 280 nm (OD 260/280), and the integrity of RNA was reflected by the 18S and 28S ribosomal bands. Two µg of RNA from each biopsy was reverse transcribed with GoScript Reverse Transcription System (Promega, USA) according to the instruction manual. Quantitative real-time PCR analysis was conducted using the CFX Real-Time PCR Detection System (Bio-Rad, USA). Each reaction contained 1 µL of resultant cDNA, 0.5 µL of each primer (10 µmol/L), 8 µL of sterile water and 10 µL of SYBR Premix Ex TaqTM (TAKARA, Japan). mRNA amplification was performed as follows: 1 min at 95 °C, then 44 cycles of 5 s at 95 °C and 30 s at 60 °C, followed by 0.5 °C increments every 5 s from 55 to 95 °C.

The primers were designed using Primer Premier 6.0 software (Premier, Canada), with the sequences as follows: omentin-1, forward 5′-GACGCCCAGAAAACAGCATC-3′, reverse 5′-CGTTGGCTGCTCTCTCGTTA-3′; adiponectin, forward 5′-CTCCTCCTCACTTCCATTCTG-3′, reverse 5′-TTTCACCGATGTCTCCCTTA-3′; β-actin, forward 5′-AGGTCATCACCATTGGCAAT-3′, reverse 5′-ACTCGTCATACTCCTGCTTG-3′. Threshold cycle (C_T_) values were obtained and relative gene expression was calculated using the formula 2^−ΔΔ CT^ with SAT fat cell fraction as a reference value.

### Immunohistochemistry

The biopsies were embedded in paraffin and cut into serial sections, and then deparaffinized and rehydrated in descending grades of alcohol, followed by staining with hematoxylin and eosin. Selected slides were incubated in 3 % H_2_O_2_ for 15 min, and then blocked with normal goat serum for 20 min. After removal of excess serum, sections were incubated with the primary antibody (Omentin-1, 1:200 dilution; Abcam, USA) at 4 °C overnight in a moisture chamber. The sections were then incubated with biotinylated secondary antibodies for 20 min followed by avidin–biotin reagents for 20 min. Slides were incubated with Diaminobenzidine (DAB) and counterstained for 1 min with hematoxylin. Light microscope observations and digital images were recorded. Positive staining for omentin-1 was brown. Expression of omentin-1 was semi-quantified by measuring the integrated optical density (IOD) of positively stained tissue via Image-Pro plus software 6.0 (Media Cybernetics, USA). The IOD of each slide was calculated from four separate fields viewed at ×200 magnification.

### Statistical analysis

Continuous data with a normal distribution were expressed as the mean ± SD or the median (lower quartile, upper quartile), as appropriate. Mean values were compared by the Student’s *t* test, while median values were compared by the Mann–Whitney U test. Categorical variables were expressed as percentages and analyzed by a Chi square test. Spearman correlation testing was performed between EAT omentin-1 mRNA levels and EAT adiponectin mRNA levels as well as serum omentin-1 levels. The associations between EAT mRNA omentin-1 levels and serum omentin-1 levels with clinical factors, including CAD and local coronary stenosis, were determined by univariate analysis and multivariate linear regression analysis. All statistical analyses were performed using SPSS 17.0 software (SPSS Inc., Chicago, IL, USA). *P* < 0.05 was considered statistically significant.

## Results

### Patient characteristics

The baseline characteristics of patients are presented in Table 1. Patients with CAD were subdivided into stenosis (n = 15) and non-stenosis (n = 13) subgroups, according to the presence or absence of local stenosis near right coronary artery ostium. Compared with the NCAD group, the CAD group were more likely to be overweight or obese, and treated with aspirin, nitrates, statins and β-blockers. Compared with the stenosis and non-stenosis subgroups, there were no significant differences in age, sex, body weight, diabetes, hypertension, current medications and laboratory examinations, except for serum creatinine levels.

**Table 1:** Baseline characteristics of patients in the CAD vs NCAD groups, and the stenosis vs non-stenosis subgroups

	CAD			NCAD (12)
	Stenosis (15)	Non-Stenosis (13)	Total (28)	
Clinical characteristics				
Age (years)	61.73 ± 4.28	60.08 ± 3.80	60.96 ± 4.08	58.50 ± 5.27
Male (%)	8 (53.3)	10 (76.9)	18 (64.3)	5 (47.1)
BMI (kg/m^2^)	25.48 ± 3.91	25.93 ± 2.82	25.69 ± 3.39	23.09 ± 2.32*^§^
Waist circumference (cm)	90.11 ± 9.68	94.15 ± 13.31	91.99 ± 11.47	81.05 ± 8.94*^§^
Hypertension (%)	4 (26.7)	3 (23.1)	7 (25.0)	1 (8.3)
T2DM (%)	8 (53.3)	8 (61.5)	16 (57.1)	3 (25.0)
Smoking (%)	9 (60.0)	9 (69.2)	18 (64.3)	4 (33.3)
LVEF (%)	57.73 ± 10.31	55.92 ± 9.51	56.89 ± 9.80	56.58 ± 7.50
Laboratory examinations				
Fasting glucose (mmol/L)	5.35 (4.98, 7.30)	5.37 (5.02, 5.65)	5.36 (5.00, 6.30)	5.28 (4.77, 6.04)
Glycosylated serum protein (%)	15.00 (14.20, 16.80)	14.50 (13.80, 17.20)	14.65 (13.88, 17.03)	14.55 (14.05, 17.75)
Fasting insulin(μU/ml)	73.10 (13.40, 104.50)	9.80 (6.05, 47.70)	16.35 (7.98, 80.88)	22.30 (11.03, 26.20)
HOMA-IR	2.49 (1.57, 6.71)	17.38 (3.82, 27.92)	4.28 (2.07, 25.75)	4.27 (2.40, 8.09)^§^
Triglycerides (mmol/L)	1.32 (0.91, 2.04)	1.71 (1.15, 2.50)	1.52 (1.02, 2.22)	1.46 (1.07, 2.59)
Total cholesterol (mmol/L)	4.25 ± 1.27	4.36 ± 0.88	4.30 ± 1.09	4.87 ± 1.32
HDL-C (mmol/L)	0.96 (0.82, 1.18)	0.81 (0.73, 1.06)	0.88 (0.77, 1.17)	1.18 (0.89, 1.73)
LDL-C (mmol/L)	2.74 ± 1.31	2.67 ± 0.93	2.70 ± 1.13	3.34 ± 0.84
Serum creatinine (μmol/L)	74.60 (65.50, 83.10)	82.10 (76.15, 93.95)^#^	78.70 (66.98, 86.60)	79.90 (65.03, 84.13)
Homocysteine (μmol/L)	15.50 (11.00, 21.90)	14.20 (11.40, 22.90)	14.35 (11.05, 21.90)	14.45 (10.93, 20.08)
hsCRP (mg/L)	1.02 (0.38, 3.85)	1.01 (0.74, 2.28)	1.02 (0.57, 3.00)	2.35 (0.67, 5.09)
BNP (pg/ml)	89.00 (43.00, 164.00)	85.00 (30.50, 169.50)	87.00 (43.00, 157.75)	165.5 (87.75, 252.25)
cTnI (μg/L)	0.01 (0, 0.02)	0.01 (0, 0.015)	0.01 (0, 0.02)	–
Medications				
Aspirin (%)	6 (40.0)	8 (61.5)	14 (50.0)	0 (0)*^§^
Nitrates (%)	13 (86.7)	13 (100)	26 (92.9)	2 (16.7)*^§^
ACEI/ARB (%)	2 (13.3)	2 (15.4)	4 (14.3)	1 (8.3)
Statins (%)	9 (60.0)	6 (46.2)	15 (53.6)	0 (0)*^§^
β-blockers (%)	9 (60.0)	11 (84.6)	20 (71.4)	0 (0)*^§^
Calcium channel blockers (%)	3 (20.0)	4 (30.8)	7 (25.0)	0 (0)
Insulin (%)	2 (13.3)	2 (15.4)	4 (14.3)	1 (8.3)
Oral hypoglycemic agents (%)	3 (20.0)	2 (15.4)	5 (17.9)	1 (8.3)

Data are shown as mean ± SD, median (lower quartile, upper quartile), or number (%)

*CAD* coronary artery disease, *NCAD* non-coronary artery disease, *BMI* body mass index, *LVEF* left ventricular ejection fraction, *HOMA-IR* homeostasis model assessment of insulin resistance, *HDL-C* high-density lipoprotein cholesterol, *LDL-C* low-density lipoprotein cholesterol, *hsCRP* high-sensitivity C-reactive protein, *BNP* brain natriuretic peptide, *cTnI* cardiac troponin I, *T2DM* type 2 diabetes, *ACEI/ARB* angiotensin-converting enzyme inhibitor/angiotensin II type 1 receptor blocker

* *P* < 0.05, CAD group vs NCAD group

^§^ *P* < 0.05, non-stenosis subgroup vs NCAD group

^#^ *P* < 0.05, stenosis subgroup vs non-stenosis subgroup

### Quantitative real-time PCR analysis

**Fig. 1:**
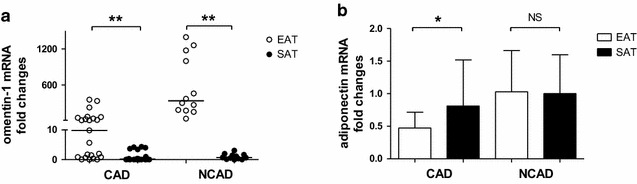
Quantitative real-time PCR analysis for adipokines in human adipose tissue. Relative omentin-1 (**a**) and adiponectin (**b**) mRNA levels in paired EAT and SAT (reference) of the two groups (CAD group, n = 23; NCAD group, n = 12). *P < 0.05, **P < 0.01. *CAD* coronary artery disease, *EAT* epicardial adipose tissue, *NS* no significant difference, *SAT* subcutaneous adipose tissue

Because of inadequate adipose tissue, three EAT and two SAT samples were excluded from the CAD group. As shown in Fig. 1a, omentin-1 mRNA levels were higher in EAT than paired SAT samples in the CAD group (9.86 vs 0.20, *P* < 0.001) and the NCAD group (334.21 vs 0.72, *P* < 0.001). Figure 1b showed that adiponectin mRNA levels were lower in EAT than paired SAT samples in the CAD group (0.47 ± 0.24 vs 0.81 ± 0.71, *P* = 0.0287), whereas adiponectin mRNA levels did not seem to differ between EAT and paired SAT samples in the NCAD group (1.03 ± 0.63 vs 1.00 ± 0.60, P = 0.89).

**Fig. 2:**
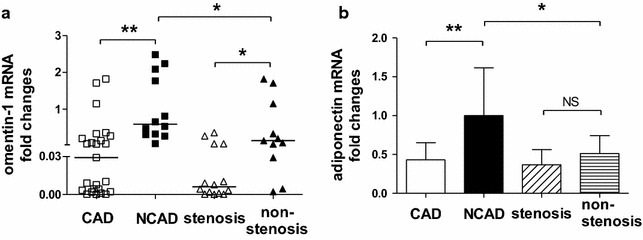
Quantitative real-time PCR analysis for adipokines in human adipose tissue. Relative omentin-1 (**a**) and adiponectin (**b**) mRNA levels in EAT of CAD group (total, n = 25; stenosis subgroup, n = 14; non-stenosis subgroup, n = 11) and NCAD group (reference, n = 12). *P < 0.05, **P < 0.01. *CAD* coronary artery disease, *EAT* epicardial adipose tissue, *NS* no significant difference, *SAT* subcutaneous adipose tissue

EAT omentin-1 and adiponectin mRNA levels were markedly decreased in CAD patients compared with NCAD patients (Fig. 2a. omentin-1 0.02 vs 0.59, *P* < 0.001; Fig. 2b. adiponectin 0.43 ± 0.22 vs 1.00 ± 0.61, *P* < 0.001). The stenosis subgroup of CAD patients had lower EAT omentin-1 expression than the non-stenosis subgroup (0.01 vs 0.14, *P* = 0.0127), while non-stenosis CAD patients had lower EAT omentin-1 expression than NCAD patients (0.14 vs 0.59, *P* = 0.0392). There seemed no significant difference between stenosis and non-stenosis subgroups of CAD patients on EAT adiponectin expression (0.37 ± 0.20 vs 0.51 ± 0.23, *P* = 0.1005), while non-stenosis CAD patients had lower EAT adiponectin expression than NCAD patients (0.51 ± 0.23 vs 1.00 ± 0.61, *P* = 0.0221).

### Immunohistochemical analysis

**Fig. 3:**
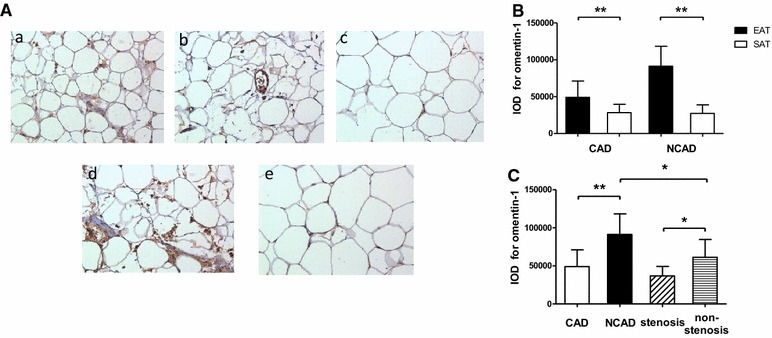
Immunohistochemical analysis for omentin-1 in human adipose tissue. **A** representative slides of adipose tissue from patients in the CAD group (Fig. 3 **A**-*a*, EAT of non-stenosis subgroup; 3**A**-*b*, EAT of stenosis subgroup; 3**A**-*c*, SAT) and the NCAD group (Fig. 3 **A**-*d*, EAT; 3**A**-*e*, SAT) (magnified ×200). **B** results of quantitative immunohistochemical analysis for omentin-1 in EAT and SAT of the two groups (CAD group, n = 9; NCAD group, n = 9). **C** results of quantitative immunohistochemical analysis for omentin-1 in EAT of CAD group (total, n = 18; stenosis subgroup, n = 9; non- stenosis subgroup, n = 9) and NCAD group (n = 9). *P < 0.05, **P < 0.01. *CAD* coronary artery disease, *EAT* epicardial adipose tissue, *SAT* subcutaneous adipose tissue, *IOD* integrated optical density

EAT and paired SAT specimens were randomly selected from the CAD group (stenosis subgroup, n = 9; non-stenosis subgroup, n = 9) and the NCAD group (n = 9), respectively. Figure 3A shows representative immuno-stained adipose sections from patients in the CAD group (Fig. 3A-a, b, c) and the NCAD group (Fig. 3A-d, e). In all patients, omentin-1 was highly expressed in EAT, most prominently in stromal vascular cells, but was barely detectable in SAT. As shown in Fig. 3B, omentin-1 protein levels were higher in EAT than paired SAT in both the CAD group (49,053 ± 22,145 vs 28,340 ± 11,314, *P* = 0.0032) and NCAD group (91,290 ± 27,006 vs 27,333 ± 11,648, *P* < 0.001). Furthermore, as shown in Fig. 3C, omentin-1 protein levels in EAT were lower in CAD patients than NCAD patients (49,053 ± 22,145 vs 91,290 ± 27,006, *P* < 0.001), while omentin-1 protein expression in EAT was lower in the stenosis subgroup than the non-stenosis subgroup (36,845 ± 12,529 vs 61,262 ± 23,447, *P* = 0.0141). In addition, EAT-derived omentin-1 protein was lower in the non-stenosis subgroup than the NCAD group (61,262 ± 23,447 vs 91,290 ± 27,006, *P* = 0.0228).

### Association of omentin-1 with coronary atherosclerosis and other clinical parameters

Table 2 showed that in univariate analysis, EAT-derived omentin-1 mRNA was negatively associated with waist circumference (beta = −0.45, 95 % CI −0.73 to −0.14), diabetes (beta = −0.36, 95 % CI −0.68 to −0.04) and CAD (beta = −0.66, 95 % CI −0.90 to −0.40). Multivariate linear regression analysis, including age, sex, waist circumference, diabetes and CAD as covariates, demonstrated that the presence of CAD was independently associated with EAT omentin-1 mRNA levels (beta = −0.57, 95 % CI −0.89 to −0.24; *P* = 0.001). Among patients with CAD, univariate analysis revealed a negative association between EAT-derived omentin-1 mRNA and local coronary stenosis (beta = −0.44, 95 % CI −0.83 to −0.05; *P* = 0.028). EAT omentin-1 mRNA expression was positively associated with that of adiponectin (r = 0.39, *P* = 0.018), but not with serum omentin-1 levels (r = 0.31, *P* = 0.062).

**Table 2:** Association between EAT as well as serum levels of omentin-1 and variables using univariate analysis and multivariate linear regression analysis

Variables	EAT mRNA levels						Serum levels					
	Univariate			Multivariate (*R* ^*2*^ = 0.419, *P* < 0.001)			Univariate			Multivariate (*R* ^*2*^ = 0.246, *P* = 0.029)		
	Beta	95 % CI	*P* value	Beta	95 % CI	*P* value	Beta	95 % CI	*P* value	Beta	95 % CI	*P* value
Age	0.01	−0.33 to 0.35	0.946	0.10	−0.19 to 0.39	0.492	−0.27	−0.58 to 0.05	0.095	−0.23	−0.53 to 0.07	0.132
Sex	0.27	−0.06 to 0.60	0.102	0.10	−0.17 to 0.37	0.463	0.30	−0.01 to 0.61	0.060	0.15	−0.12 to 0.42	0.272
WC	−0.45	−0.73 to −0.14	0.005	−0.12	−0.42 to 0.19	0.445	−0.36	−0.66 to −0.05	0.024	−0.16	−0.47 to 0.15	0.308
CAD	−0.66	−0.90 to −0.40	<0.001	−0.57	−0.89 to −0.24	0.001	−0.59	−0.86 to −0.33	<0.001	−0.35	−0.67 to −0.03	0.036
T2DM	−0.36	−0.68 to −0.04	0.029	−0.11	−0.39 to 0.17	0.438	−0.29	−0.60 to 0.03	0.072			
TC	−0.11	−0.24 to 0.47	0.520				0.36	0.05 to 0.66	0.024	0.23	−0.04 to 0.50	0.093
HTN	−0.26	−0.57 to 0.07	0.127				−0.32	−0.63 to −0.01	0.046	−0.02	−0.35 to −0.31	0.887
HOMA-IR	−0.21	−0.53 to 0.12	0.210				−0.32	−0.64 to −0.01	0.041	−0.15	−0.48 to 0.17	0.350

Multivariate linear regression model includes age, sex and other variables with P < 0.05 in univariate analysis

*EAT* epicardial adipose tissue, *CI* confidence interval, *WC* waist circumference, *CAD* coronary artery disease, *TC* triglycerides, *HTN* hypertension, *T2DM* type 2 diabetes, *HOMA-IR* homeostasis model assessment of insulin resistance

Omentin-1 levels in serum were lower in the CAD group than the NCAD group (373.71 vs 659.39 ng/mL, *P* < 0.001). As shown in Table 2, in univariate analysis, serum omentin-1 levels were negatively associated with waist circumference (beta = −0.36, 95 % CI −0.66 to −0.05), HOMA-IR (beta = −0.32, 95 % CI −0.64 to −0.01), hypertension (beta = −0.32, 95 % CI −0.63 to −0.01) and CAD (beta = −0.59, 95 % CI −0.86 to −0.33). A multivariate linear regression analysis model, including age, sex, waist circumference, HOMO-IR, hypertension, triglycerides and CAD, demonstrated that the presence of CAD was independently associated with serum omenitn-1 levels (beta = −0.35, 95 % CI −0.67 to −0.03; *P* = 0.036).

Adiponectin levels in serum were lower in the CAD group than the NCAD group (4.57 ± 0.99 vs 6.98 ± 1.11 ng/mL, *P* < 0.001). As shown in Table 3, multivariate linear regression analysis indicated that the presence of CAD was independently associated with EAT adiponectin mRNA levels (beta = −0.49, 95 % CI −0.81 to −0.14; *P* = 0.006) and serum adiopnectin levels (beta = −0.82, 95 % CI −0.99 to −0.66; *P* < 0.001).

**Table 3:** Association between EAT as well as serum levels of adiponectin and variables using univariate analysis and multivariate linear regression analysis

Variables	EAT mRNA levels						Serum levels					
	Univariate			Multivariate (*R* ^*2*^ = 0.246, *P* = 0.01)			Univariate			Multivariate (*R* ^*2*^ = 0.799, *P* < 0.001)		
	Beta	95 % CI	*P* value	Beta	95 % CI	*P* value	Beta	95 % CI	*P* value	Beta	95 % CI	*P* value
Age	−0.20	−0.53 to 0.13	0.231	−0.09	−0.39 to 0.22	0.557	−0.25	−0.57 to 0.07	0.117	0.02	−0.13 to 0.18	0.767
Sex	0.16	−0.18 to 0.50	0.338	0.05	−0.26 to 0.35	0.770	0.05	−0.28 to 0.38	0.752	−0.07	−0.22 to 0.08	0.351
BMI	−0.32	−0.65 to 0.001	0.050	−0.10	−0.43 to 0.23	0.526	−0.23	−0.55 to −0.09	0.151	−0.18	−0.35 to −0.01	0.035
CAD	−0.56	−0.83 to −0.27	<0.001	−0.49	−0.81 to −0.14	0.006	−0.74	−0.96 to −0.52	<0.001	−0.82	−0.99 to −0.66	<0.001
TC	−0.03	−0.39 to 0.33	0.861				−0.32	−0.63 to −0.01	0.044	−0.57	−0.73 to −0.41	<0.001
HOMA-IR -0.24	−0.24	−0.56 to 0.09	0.148				−0.23	−0.55 to 0.10	0.163			
HTN	−0.26	−0.58 to 0.07	0.117				−0.09	−0.42 to 0.24	0.578			
Glucose	−0.18	−0.50 to 0.16	0.293				0.03	−0.30 to 0.36	0.856			

Multivariate linear regression model includes age, sex and other variables with P < 0.05 in univariate analysis

*EAT* epicardial adipose tissue, *CI* confidence interval, *BMI* body mass index, *CAD* coronary artery disease, *HOMA-IR* homeostasis model assessment of insulin resistance, *HTN* hypertension, *TC* triglycerides

## Discussion

In this study, we found that in patients with CAD, serum and EAT omentin-1 levels were markedly lower than in patients without CAD, and this inverse association remained after adjusting for well-known risk factors. Furthermore, patients with CAD had lower omentin-1 expression in EAT surrounding stenotic coronary segments compared with normal coronary segments. Watanabe and co-workers using immunohistochemistry showed that patients with CAD had substantially lower omentin-1 expression in EAT and coronary endothelium [20]. However, the baseline characteristics of CAD and NCAD patients in Watanabe’s study were not well matched. Our present study complements and extends prior findings by revealing that omentin-1 expression in EAT of CAD patients was significantly lower on both the mRNA and protein levels, and these inverse associations between CAD and EAT omentin-1 mRNA as well as protein expression remained after adjusting for well-known risk factors. These findings suggest that omentin-1 derived from EAT associates with CAD, or in another way, the entirely coronary atherosclerosis.

However, whether EAT omentin-1 expression associated with local coronary atherosclerosis has not been clarified. Recent researches indicate that EAT or perivascular adipose tissue has crucial roles in the maintenance of cardiovascular homeostasis and remodeling under physiological or pathological circumstances [21]. It has been established that EAT is a source of adipocytokines, and is closely linked to the initiation and progression of atherosclerosis [4, 5]. More recently, Benedicte et al. identified 400 common genes involved in extracellular matrix remodeling, thrombosis and inflammation, which were expressed in EAT from the periatrial region, periventricular area and surrounding the pericoronary artery. The authors also showed that omentin was the most highly upregulated gene in EAT compared with SAT [22]. Similarly, we found that omentin-1 mRNA and protein levels were significantly higher in EAT than SAT, regardless of pre-existing CAD. Considering the contiguous anatomic location and microcirculatory connection, it is reasonable to speculate that tissue-specific expression of omentin-1 in EAT closely relates to local coronary atherosclerosis via paracrine and vasocrine mechanisms.

To examined the association between adipocytokine expression in EAT and local coronary atherosclerosis, Verhagen et al. obtained perivascular adipose tissue near to stenotic and non-stenotic coronary artery segments from the same CAD patient undergoing CABG, and he revealed a negative relationship between EAT-derived adipocytokine production and local coronary atherosclerosis [23]. Likewise, we found that among patients with CAD, omentin-1 mRNA and protein levels in EAT surrounding coronary artery stenotic segments were markedly decreased. There was a negative association between EAT-derived omentin-1 mRNA and local coronary stenosis among patients with CAD in a univariate analysis. Therefore, we presume that omentin-1 mRNA expression in EAT was inversely associated with entirely and local coronary atherosclerosis. The former association was further investigated in the comparison of the non-stenosis CAD patients and NCAD patients showing that omentin-1 mRNA and protein levels were lower in the non-stenosis subgroup, which indicated that the negative association between omentin-1 expression in EAT and CAD is independent of local coronary atherosclerosis.

Generally, explanation of the association between EAT derived adipocytokines and coronary atherosclerosis is oversimplified and paracrine effects of EAT may be overestimated. In fact, the delicate equilibrium between pro-inflammatory and anti-inflammatory adipocytokines is complicated and susceptible to disruption in pathological conditions [7]. Adiponectin, positively associated with omentin-1 in EAT mRNA expression in our study, was found to be lower in patients with CAD on EAT mRNA levels and serum levels [5, 24], moreover, we observed that this reverse association between EAT mRNA levels as well as serum levels of adiponectin and CAD still existed adjusting for traditional risk factors. However, adiponectin mRNA expression was not expectantly significantly lower in EAT near to stenotic segments compared with non- stenotic segments in both Verhagen’s study and this study. In another small-scale study, adiponectin mRNA levels were not significantly lower in EAT near to coronary segments containing a bare metal stent with heavier atherosclerotic burden when compared with EAT adjacent to segments without a stent [25]. Moreover, secretion of pro-inflammatory adipocytokines (IL-1α, IL-17, IL-18 and IL-23) from EAT adjacent to coronary stenotic segments was markedly reduced in Verhagen’s study [23]. Overall, patients with CAD have a stronger pro-inflammatory profile of adipocytokines in EAT than NCAD patients [26], whereas the regulation of adipocytokines in EAT derived from coronary stenotic segments of advanced CAD patients appears to be sophisticated.

Omentin-1 is secreted by visceral fat, and its local concentration in visceral fat may greatly exceed that in the circulation or SAT [27]. In this study, EAT omentin-1 mRNA expression was not positively associated with serum omentin-1 levels. Meanwhile, because omentin-1 circulates in blood, it may improve insulin sensitivity and glucose metabolism in the entire body, which would have an impact on the initiation and progression of obesity-related disorders such as CAD. In the present study, omentin-1 levels in serum were negatively associated with the presence of CAD, even after adjusting for traditional risk factors. This relationship was confirmed in a meta-analysis demonstrating that serum omentin-1 levels were independently and negatively associated with CAD [28]. The present study also showed that serum omentin-1 was negatively correlated with waist circumference and HOMA-IR. These results are in agreement with findings that serum omentin-1 levels are increased in most obesity following bariatric surgery with lower cardiovascular risk [29] and in diabetes after treatment of pioglitazone with improved cardiac diastolic function [30].

The in vivo effect of omentin-1 halted the development of atherosclerosis has been investigated in apolipoprotein E-deficient (*Apoe*^−*/*−^) mice with decreased macrophage infiltration and pro-inflammatory genes expression [20, 31]. *In vitro*, omentin-1 promoted differentiation of macrophages into the anti-inflammatory M2 phenotype, and suppressed inflammatory responses and foam cell formation [20]. Omentin-1 has been demonstrated to promote vasodilation and survival of endothelial cells through activation of AMPK/eNOS pathways [32], and to alleviate inflammation in endothelial cells via inhibition of TNF-α pathways [33]. In addition, omentin-1 inhibited monocyte adhesion to smooth muscle cells by reducing VCAM-1 expression through inhibition of p38/JNK signaling [34]. Furthermore, omentin-1 was observed to increase in macrophage-derived foam cells and medial layer VSMCs within advanced coronary plaques and the circulating blood in patients with acute coronary syndrome (ACS) [20], this implies that omentin-1 may exert cardio-protective effects in the acute-phase of ACS, which is of significant importance for targeted therapy.

The limitations of this study should be noted. The characteristics of patients in the CAD and NCAD groups were not balanced, which may confound the association between omentin-1 expression and CAD. Although multivariate regression analysis was performed, the existence of residual confounding variables cannot be excluded. Because of the small sample size, it was not possible to investigate the inverse association between EAT omentin-1 levels and local coronary atherosclerosis in CAD patients by multivariate linear regression analysis. This was a cross-sectional and observational study, therefore, the results should be considered exploratory and hypothesis generating. Conclusions concerning the mechanism and causality between omentin-1 and coronary atherosclerosis cannot be made. Further studies are required to evaluate whether EAT-derived omentin-1 has a direct effect on coronary atherosclerosis.

## Conclusions

This study established that EAT and serum omentin-1 levels were negatively associated with the presence of CAD. However, the inverse relationship between EAT omentin-1 levels and local coronary atherosclerosis requires further elucidation. Future studies focusing on the causality between EAT omentin-1 expression and coronary atherosclerosis as well as its underlying mechanisms are warranted.

## Abbreviations

EAT: epicardial adipose tissue
SAT: subcutaneous adipose tissue
CAD: coronary artery disease
NCAD: non-coronary artery disease
T2DM: type 2 diabetes mellitus
CABG: coronary artery bypass grafting
BMI: body mass index
hsCRP: high-sensitivity C-reactive protein
HOMA-IR: homeostasis model assessment of insulin resistance
ELISA: enzyme-linked immunosorbent assay
C_T_: threshold cycle
DAB: diaminobenzidine
IOD: integrated optical density
*Apoe*-*/*-: apolipoprotein E-deficient
ACS: acute coronary syndrome
